# The occupational sitting and physical activity questionnaire (OSPAQ): a validation study with accelerometer-assessed measures

**DOI:** 10.1186/s12889-020-09180-9

**Published:** 2020-07-06

**Authors:** Iris Maes, Margo Ketels, Delfien Van Dyck, Els Clays

**Affiliations:** 1 Department of Movement and Sports Sciences, Faculty of Medicine and Health Sciences, Ghent University, Ghent, Belgium; 2 Department of Public Health and Primary Care, Faculty of Medicine and Health Sciences, Ghent University, University Hospital Ghent, entrance 42 (4K3), Corneel Heymanslaan 10, 9000 Ghent, Belgium

**Keywords:** Occupational physical activity, Validity, OSPAQ, Accelerometer, Physically active professions, Sedentary professions

## Abstract

**Background:**

The Occupational Sitting and Physical Activity Questionnaire (OSPAQ) was developed as an easy-to-use instrument for self-reported assessment of percentage sitting, standing, walking, and performing heavy labour in a workplace setting. This study aimed to evaluate the concurrent validity of all dimensions of the OSPAQ compared to accelerometer-assessed measures of occupational physical activities in a mixed sample of sedentary and physically active professions.

**Methods:**

Data from the Flemish Employees’ Physical Activity (FEPA) study were used, including employees from the service and production sector. All participants filled in a questionnaire, underwent clinical measurements, and wore two Axivity AX3 accelerometers for at least 2 consecutive working days. Intraclass (ICC) and Spearman rho correlations (r) were analyzed to assess concurrent validity.

**Results:**

The sample included 401 workers (16% sedentary profession) with a mean age of 39.2 (± 11) years. Concurrent validity was good and moderate for assessing percentage of sitting (ICC = 0.84; *r* = 0.53), and standing (ICC = 0.64; *r* = 0.53), respectively. The concurrent validity for walking was weak to moderate (ICC = 0.50; *r* = 0.49), and weak for performing heavy labour (ICC = 0.28; *r* = 0.35). Stronger validity scores were found in sedentary professions for occupational sitting and standing. In physically active professions, an underestimation of self-reported sitting and standing was found, and an overestimation of self-reported walking and heavy labour. No significant self-reported over- or underestimation was found for sitting and heavy labour in sedentary professions, but an underestimation of self-reported standing and an overestimation of self-reported walking was observed.

**Conclusions:**

The OSPAQ has acceptable measurement properties for assessing occupational sitting and standing. Accelerometer-assessed measures of occupational walking and heavy labour are recommended, since a poor concurrent validity was found for both.

## Background

The contribution of physical activity (PA) to good health is widely documented in the literature [1]. PA has a particularly beneficial role in the prevention of cardiovascular diseases (CVD) and metabolic conditions such as obesity and type two diabetes mellitus [2, 3]. Nevertheless, the positive health effects differ depending on the domain of PA. In contrast to leisure time physical activity (LTPA), occupational physical activity (OPA), including heavy labour tasks such as carrying and lifting, increases the risk of CVD [2, 4, 5], mortality [6], and long-term sickness absence [7]. These contrasting health effects of LTPA and OPA are known in the literature as “the PA health paradox” [8]. Furthermore, several studies have consistently shown that sedentary behavior (SB) is associated with all-cause and cardiovascular mortality [9, 10], independent from the level of PA. Office based professions in which people are sitting down for a long period result in a greater risk of adverse outcomes, for physical as well as mental health [11, 12]. Lastly, increasing evidence supports the finding that participating several years in shift work schedules is associated with adverse health consequences compared to participating in day work schedules [13, 14]. Also, shift workers are claimed to engage less in moderate-vigorous PA and to have longer periods of sitting time compared to those who work during day time [15]. However, evidence is scarce and exploring the OPA patterns in both day and shift workers is highly recommended. Because of the detrimental health effects of OPA and SB, interventions at the workplace among workers with different job patterns are required to change these behaviors. In this context, OPA and SB need to be measured properly.

Valid and reliable instruments are necessary to determine the type and amount of OPA and SB [16]. Accelerometer-assessed measures as well as self-reported measures can be used to assess SB and PA, and both have specific advantages and disadvantages. Accelerometer-assessed measures provide information about the amount, frequency, and duration of PA during day- and nighttime through activity patterns and activity intensities [17]. It is recommended to use accelerometers in combination with a diary, since accelerometers are not able to measure the context in which individuals perform PA. Accelerometers, in combination with a diary, have been frequently used to assess SB and PA in the work context [18, 19]. However, using accelerometers in research is time consuming and expensive. Consequently, only a limited number of people can be reached when using accelerometers. Self-reported measures like questionnaires on the other hand, are less expensive and easy to use in larger study samples. Nonetheless, these self-reported measures also have a number of shortcomings, such as a limited reliability and validity due to social desirability, over- or underestimation, and recall bias [20, 21].

The Occupational Sitting and Physical Activity Questionnaire (OSPAQ) has been developed to assess occupational sitting, standing, walking, and performing heavy labour based on percentage of time spent in those activities at work [22]. It is a low-cost and easy-to-use instrument, and manageable on a large scale. A couple of studies have been carried out to validate the OSPAQ, but results are scarce and sometimes even contradictory. Additionally, previous studies were conducted in office based professions. A few validation studies [19, 22, 23], which used ActiGraph GT3X+ accelerometers, Actigraph GT1M accelerometers, and activPAL activity monitors respectively, reported a moderate to strong validity of the OSPAQ for estimating occupational sitting and standing, and suggested that the OSPAQ is suitable for application in a broad range of office based professions. However, these findings were not confirmed by a number of other studies. The study of van Nassau and colleagues [24] showed low validity (*r* = 0.35 to 0.48) for occupational sitting and inconsistent findings (*r* = 0.16 to 0.68) for occupational standing in an office based sample using an activPAL3 activity monitor and ActiGraph GT1M and GT3X accelerometers. Poor validity was found for walking in several studies performed in an office based sample which used respectively an activPAL activity monitor and an ActiGraph wGT3X-BT accelerometer [23, 25]. In general, an underestimation of self-reported standing and sitting was found, whereas an overestimation of self-reported walking during work was reported [25]. The validity of the OSPAQ for assessing heavy labour has not been examined yet in previous studies. Furthermore, previous validation studies examined small to medium sized study populations, which increases the margin of error.

In summary, even though a few studies reported moderate to high validity coefficients for occupational sitting in mainly office-based professions, the validity of the OSPAQ is questionable for occupational standing, walking, and heavy labour in office based professions [23]. Up till now, no validation data are available for non-office based professions (i.e. physically more active jobs). To address the shortcomings of previous research, this study aimed to evaluate the concurrent validity, a type of criterion-related validity, of all dimensions (i.e. sitting, standing, walking, and heavy labour) of the OSPAQ compared to accelerometer-assessed measures of OPA in a mixed sample of sedentary and physically active professions.

## Method

### Study population and design

Data from the FEPA (Flemish Employees’ Physical Activity) study were used, this study has been described in detail elsewhere [26]. In short, participants were recruited by means of convenience sampling across seven different companies within the service and manufacturing sector. The included companies approved that the data collection could be carried out at the workplace during working hours. Inclusion criteria for the workers were non-pregnancy, no exclusive night workers, employment rate of at least 50%, and a sufficient knowledge of the Dutch language. In total 1135 eligible workers were contacted and invited to participate in the study. Of this sample, 430 workers (response rate of 38%) were willing to participate voluntarily and signed an informed consent. Data were collected from February 2017 until June 2018. Eventually, 29 participants did not fill in the questionnaire and/or did not participate in the accelerometer-assessed measures due to drop-out or technical issues, which leads to a final sample of 401 participants. The final sample included both workers engaged in physically active work (i.e. employees working in health care, manufacturing, food, and plastic sector) and workers primarily engaged in sedentary work (i.e. administrative professions). A more detailed overview of the flow of the recruitment of the study population is shown in an additional figure [see Additional file 1]. The FEPA study was approved by the Research Ethical Committee of Ghent University Hospital (number 2017/0129).

### Procedure

After receiving a study invitation letter, interested participants were asked to complete a written informed consent form, to fill in a self-reported questionnaire including the OSPAQ (paper or online version) and to hand it over at the clinical screening appointment. A trained researcher conducted the clinical screening at the workplace of the participants during working hours and attached two accelerometers that were worn for at least 2 consecutive working days, 24-h/day. More specific, the participants were asked to wear the devices on typical/normal working days. During this recording time, the participants were asked to fill in a paper-based diary to report their daily routines (time intervals of work, leisure time, bedtime, and non-wear time). Additionally, they had to report daily the time at which they performed the reference measurement in order to calibrate the accelerometers (15 s of standing still in an upright and neutral position). After the accelerometer measurement period of at least 2 consecutive typical working days the participants returned their equipment and diary to the researcher.

### Measurements

#### Questionnaire

The questionnaire included information about participants’ socio-demographic situation (e.g. age, sex, education level), working conditions (e.g. specific type of profession, seniority, work schedule, working hours), and different activities during working hours. SB and PA during working hours were captured by the Occupational Sitting and Physical Activity Questionnaire (OSPAQ), a self-reported instrument to assess the percentage of the occupational time spent sitting, standing, walking, and the time spent doing heavy labour [22]. Participants were asked to indicate the percentage of time spent on those activities during working hours on a typical working day. The participants received a slightly adjusted instruction to fill in the OSPAQ compared to other studies, namely to keep in mind a typical working day instead of the last seven working days (i.e. “How would you describe a typical working day in your current job? This only includes your working day. Commuting and everything outside the work setting is not included.”). So when accumulating all percentages a total percentage of 100 was obtained: for example, 55% occupational sitting, 25% walking, 15% standing, and 5% performing heavy labour [22].

#### Clinical screening

Body mass index (BMI) calculations were based on measurements of height and weight by using the Seca 704 column scale (SECA Medical Measuring Systems and Scales, Birmingham, UK, scales 701/704). The participants were asked to remove heavy outer garments, e.g. jackets, heavy sweaters, belts, watches, and other belongings in their work clothes/pockets, and their shoes before standing on the scale. A tapeline was used to measure the waist circumference, which was defined as the narrowest point between the lowest rib and the iliac crest.

#### Accelerometer-assessed measures

The eligible participants were asked to wear two Axivity AX3 accelerometers to measure SB and PA, one placed on the middle of the back and one on the right thigh. Synchronisation between the two accelerometers was achieved by the initialisation procedure; both accelerometers were started simultaneously and linked to the real-time clock of the computer by using the internal timer of the accelerometers [27]. Both accelerometers were oriented with the x-axis and the USB port facing down, y-axis horizontally to the left, and the z-axis horizontally forward. Fixation of the accelerometers on the skin was realised with Opsite Flexifit wound foil. For the data analysis, a custom-made MATLAB software named Acti4 was used to determine the type and duration of each activity (The National Research Centre for the Working Environment, Copenhagen, Denmark and Federal Institute for Occupational Safety and Health, Berlin, Germany) [27]. In order to obtain data of OPA, the Acti4 software [28] divided the accelerometer data into intervals (i.e. work, leisure time, and sleep intervals) based on the information found in the paper-based diaries. Intervals were identified as non-wear time if (a) the Acti4 software showed no movement for more than 90 min, (b) the participants reported ‘non-wear periods’ in their diary, or (c) artefacts or missing data were detected by the Acti4 software. A work interval was considered to be valid if it comprises more than 4 h/day of accelerometer wear-time or more than 75% of the individual’s average reported wear-time across days. Data on a daily basis were only included in the data pool if a minimum of 10 h of accelerometer data was available. Only participants with at least 1 valid working day of accelerometer data were included for further analyses.

### Data and statistical analyses

The percentage of time spent in occupational sitting, standing, and walking was estimated based on accelerometer data by dividing the total measured hours of each activity during working hours by the total accelerometer-assessed amount of time spent at work. The different occupational activities were then expressed as the percentage of total time at work. The total amount of occupational heavy labour was calculated by the sum of cycling, fast walking (> 1.67 steps/sec), walking on stairs, and running during working hours. The sum of the previous activities was then divided by the total accelerometer-assessed time of working hours. Heavy labour is then also expressed as the percentage of total time at work.

Statistical analyses were conducted in SPSS Statistics, Version 25.0 (SPSS Inc., Chicago, Illinois) and the level of significance was set at *p* < 0.05 (5%). Descriptive statistics were computed for all variables and shown as mean (Standard Deviation (SD)) and as percentage of occupational time. Differences in baseline characteristics between job types were assessed using independent samples t-tests (quantitative variables) and chi-quare tests (qualitative variables). Differences between accelerometer-assessed measures and self-reported measures were calculated using paired samples t-tests and differences in mean values between physically active and sedentary jobs were calculated using independent samples t-tests. To compare the items of the OSPAQ questionnaire with the accelerometer-assessed measured amount of occupational sitting, standing, walking, and heavy labour, intraclass correlation coefficients (ICC) were calculated. ICCs were interpreted as weak (< 0.50), low (0.50–0.59), moderate (0.60–0.69), or strong (≥0.70) [22]. Spearman correlations were used to verify the concurrent validity of the OSPAQ occupational sitting, standing, walking, and heavy labour items by comparing the questionnaire items with accelerometer-assessed values. The strength of the Spearman correlation was interpreted as weak (< 0.30), low (0.30–0.49), moderate (0.50–0.69), strong (0.70–0.89), or very strong (> 0.90) [29]. Subgroup analyses were done for type of profession (sedentary professions versus physically active professions). The possible moderating effect of the work schedule, i.e. day or shift work, on the association between self-reported and accelerometer-assessed measures was tested in a multiple linear regression analysis for each type of behaviour separately. The accelerometer-assessed measure was included as the dependent variable and the interaction term (work schedule multiplied by self-reported measure) along with both main effects was included in the analysis as independent variable. In case statistically significant moderating effects were observed, the concurrent validity was analysed separately for day and shift workers.

## Results

The sample included 401 workers (58% female), aged between 20 and 65 years (mean 39.2; SD 11). A high educational level was obtained by 53% of the participants. Almost 60% worked in shifts and 84% had a physically active profession. The participants wore their accelerometers for an average of 2.7 days (± 0.9), with an average working day of 7.9 h (± 1.2). More detailed baseline characteristics are provided in Table 1. A comparison based on job type showed that workers with sedentary jobs were significantly more highly educated, had more years of seniority, worked less in shifts, smoked less, and had less accelerometer-assessed working hours compared to workers involved in physically active jobs. Workers with physically active jobs and sedentary jobs were comparable regarding sex, age, workhours per week, BMI, and average accelerometer-assessed wear time (Table 1).

**Table 1:** Baseline characteristics of the participants

Characteristics	Total sample	Physically active jobs	Sedentary jobs	Difference
	Mean (SD) or % (n/N)	Mean (SD) or % (n/N)	Mean (SD) or % (n/N)	*P*-value
**Number of participants**	401	83.6 (331/396)	16.4 (65/396)	
**Sex (% women)**	58.4 (234/401)	57.1 (189/331)	64.6 (42/65)	0.28
**Age (years)**	39.2 (11.0)	38.8 (11.2)	40.9 (10.3)	0.16
**Educational level (%)**				**< 0.001**
Lowª	15.5 (62/401)	18.8 (59/331)	1.5 (1/65)	
Mediumª	31.7 (127/401)	32.9 (109/331)	24.6 (16/65)	
Highª	52.9 (212/401)	49.2 (163/331)	73.8 (48/65)	
**Seniority (%)**				**0.03**
Up to 5 years	47 (188/400)	49.6 (164/331)	33.9 (22/65)	
> 5 years	53 (212/400)	50.4 (167/331)	66.1 (43/65)	
**Work schedule (%)**				**< 0.001**
Day work	41.6 (167/399)	35.6 (118/329)	72.3 (47/65)	
Shift work	57.9 (232/399)	63.7 (211/329)	27.7 (18/65)	
**Workhours per week**	37.0 (6.57)	36.7 (6.31)	38.4 (7.70)	0.06
**Current smokers (%)**				**0.04**
Yes	22.1 (310/398)	23.9 (79/331)	12.3 (8/65)	
No	77.9 (88/398)	76.1 (252/331)	87.7 (57/65)	
**BMI group (%)**				0.42
Underweight (BMI < 18.5)	1.2 (5/401)	1.5 (5/331)	0 (0/65)	
Normal weight (BMI < 25)	57.1 (229/401)	56.2 (186/331)	64.6 (42/65)	
Overweight (BMI 25–30)	31.7 (127/401)	31.7 (105/331)	29.2 (19/65)	
Obese (BMI ≥ 30)	10 (40/401)	10.6 (35/331)	6.2 (4/65)	
**Accelerometry**				
Work (h/day)	7.9 (1.2)	7.9 (1.2)	7.7 (1.2)	**0.02**
Wear time (days)	2.7 (0.9)	2.7 (1.0)	3.0 (0.8)	0.29

Data are presented as mean ± SD or % (n/N) . *BMI* body mass index, *h* hours. ªEducational level: until primary school as “low”, secondary school and/or 1 to 2 years of specialization as “medium”, and university or university college as “high”

Table 2 shows the mean percentages of work time for self-reported and accelerometer-assessed measures of occupational sitting, standing, walking, and heavy labour of the total sample, and separately for participants with physically active professions and those with sedentary professions. The dataset, including the job type of the participants, self-reported and accelerometer-assessed measures of sitting, standing, walking, and heavy labour are provided in an additional file [see Additional file 2]. The differences between both types of profession are presented in Table 2, as well as the differences between self-reported and accelerometer-assessed measures. In the total sample, participants underestimated self-reported sitting and standing (*p* < 0.001), whereas self-reported walking and heavy labour were significantly overestimated (*p* < 0.001). Similar results appear for workers with physically active professions, whereas no significant self-reported over- or underestimation was found for occupational sitting (*p* = 0.35) and heavy labour (*p* = 0.09) in the participants with a sedentary profession. For participants with physically active jobs the percentage of each activity, both self-reported and accelerometer-assessed measures, was significantly different compared to the percentages of participants with sedentary professions.

**Table 2:** Percentages of self-reported (OSPAQ) and accelerometer-assessed measures of different occupational activities.

	Total sample (401)	Physically active jobs (331^a^)	Sedentary jobs (65^a^)	*P*-value
% Sitting: mean (SD)				
OSPAQ	26.34 (29.55)	20.20 (25.55)	57.77 (32.73)	**< 0.001**
Accelerometers	34.34 (23.69)	30.40 (21.02)	55.30 (25.61)	**< 0.001**
	**< 0.001**	**< 0.001**	0.35	
% Standing: mean (SD)				
OSPAQ	28.14 (22.71)	29.82 (22.85)	18.63 (18.34)	**< 0.001**
Accelerometers	35.38 (17.06)	37.01 (15.93)	27.19 (19.81)	**< 0.001**
	**< 0.001**	**< 0.001**	**< 0.001**	
% Walking: mean (SD)				
OSPAQ	29.90 (18.64)	31.26 (18.46)	17 (13.90)	**< 0.001**
Accelerometers	15.55 (8.36)	16.73 (8.45)	9.78 (4.94)	**< 0.001**
	**< 0.001**	**< 0.001**	**< 0.001**	
% Heavy labour: mean (SD)				
OSPAQ	16.68 (18.22)	18.91 (18.64)	6.40 (11.79)	**< 0.001**
Accelerometers	13.53 (7.22)	14.45 (7.36)	8.83 (4.43)	**< 0.001**
	**0.002**	**< 0.001**	0.09	

Data are presented as mean ± SD. ^a^ = 5 missings

### Concurrent validity

Spearman correlations (r) and intraclass correlations (ICC) were calculated between self-reported and accelerometer-assessed measures (Table 3). For the whole sample, a good concurrent validity was obtained for assessing percentage of sitting (ICC 0.84; Spearman rho 0.69), and a moderate concurrent validity for the proportion of time spent standing (ICC 0.64; Spearman rho 0.53). The concurrent validity for the whole sample for walking was weak to moderate (ICC 0.50; Spearman rho 0.49), and weak for heavy labour (ICC 0.28; Spearman rho 0.35). In general, similar patterns were observed for physically active professions as well as sedentary professions. However, participants with sedentary professions showed stronger validity scores for occupational sitting and standing compared to those with physically active professions. Weak to low concurrent validity were obtained for walking and heavy labour in both types of profession. A moderation effect of the work schedule of the workers, i.e. shift or day work, on the association between self-reported and accelerometer-assessed occupational activities was observed for sitting (*p* < 0.05) and standing (*p* < 0.05) for the whole sample, which was not the case for walking (*p* = 0.13) and heavy labour (*p* = 0.40). The concurrent validity was noticeably lower in shift workers compared to day workers. Results of the stratified analysis are presented in additional file 3 [see Additional file 3].

**Table 3:** Concurrent validity of the OSPAQ for measuring occupational activities compared with accelerometer-assessed measures

	Total sample (401)		Physically active jobs (331^a^)		Sedentary jobs (65^a^)	
	r	ICC	r	ICC	r	ICC
% Sitting	0.69	0.84	0.63	0.81	0.70	0.81
% Standing	0.53	0.64	0.47	0.61	0.66	0.75
% Walking	0.49	0.50	0.43	0.46	0.48	0.42
% Heavy labour	0.35	0.28	0.25	0.20	0.49	0.32

Spearman correlations = r; Intraclass correlations = ICC; ^a^ = 5 missings

Bland-Altman plots [30] are shown in additional file 4 [see Additional file 4]. The plots were applied to test the agreement between the time spent in each activity, i.e. sitting, standing, walking, and heavy labour, measured by the OSPAQ and accelerometers among physical active and sedentary professions. The plots show the difference of the two paired measurements, e.g. time spent sitting from the OSPAQ and from accelerometers, plotted against the mean of the two measurements.

## Discussion

The aim of the current study was to validate the self-reported Occupational Sitting and Physical Activity Questionnaire (OSPAQ) against accelerometer-assessed measures of occupational sitting and physical activities, in a mixed sample of sedentary and physically active workers. This research was conducted in order to legitimize the use of the OSPAQ in different profession types.

### Concurrent validity

The results of the current study showed that the OSPAQ is a valid instrument to assess time spent sitting at work in both sedentary and physically active professions. These results are in line with other studies, which observed moderate to strong correlations for sitting in office based workers [19], but also in a mixed sample of workers with different occupational activity levels [22]. Although the time spent sitting was significantly lower in the physically active professions, it seemed that both participants with physically active professions as well as participants with sedentary professions were able to accurately assess the percentage of their sitting time at work. A possible explanation for the good validity of measuring time spent sitting might be that sitting is a more habitual and/or routine activity compared to lifting or walking on stairs which are less structured activities [31]. The study of Marshall and colleagues [31] also confirmed that time spent sitting during work is more accurately recalled than sitting for leisure activities. Besides the validity of sitting, our findings showed that the percentage of time spent sitting at work amounted more than 50% in sedentary jobs, which increases the risk for chronic health problems and calls for further action. Considering the large amount of occupational sitting and given the high validity of sitting assessed with the OSPAQ, this questionnaire might be an important contribution to elucidate the relation between sitting time and health outcomes in studies with large sample sizes.

For the total sample, the OSPAQ showed to be a moderately valid tool to assess occupational standing. Nevertheless, in subgroup analyses we found a strikingly higher validity for standing in sedentary professions compared to the physically active professions. These findings are comparable to other studies which reported moderate associations for the measurements of standing for office based workers [19]. A possible explanation for this finding can be that workers with a sedentary profession are more aware of their standing behaviour, due to the very noticeable difference between sitting and standing, whereas standing in physically active professions is more integrated in the daily activities, which makes it more difficult to differentiate standing from other activities.

The concurrent validity of time spent walking and performing heavy labour at work in this study was low to weak. Subgroup analyses showed that the validity for measuring heavy labour is even lower for physically active professions compared to sedentary professions (*r* = 0.25 vs. 0.49). The lack of agreement between the OSPAQ and accelerometer-assessed measurements for walking is similar to the findings reported by Chau and colleagues [22] (*r* = 0.29) and Pedersen and colleagues [23] (*r* = 0.54) in an office based sample. Walking might be more integrated in the daily occupational activities, which makes it difficult for workers to accurately define the amount of time spent walking in percentage.

Our study showed the lowest validity for performing heavy labour, both in sedentary professions as well as in physically active professions. A possible explanation might be that heavy labour, consisting of physically active tasks, is less structured and more spread over the day compared to sitting or standing. This might make it difficult to accurately define the amount of time performing heavy labour in percentages. Another possible explanation for the low validity of performing heavy labour at work is related to using accelerometers to measure heavy labour. Accelerometers are generally recognised as a good method for determining concurrent validity. However, accelerometers are not capable of detecting the intensity of upper body movements, such as heavy lifting and carrying [28], which are important aspects of heavy labour. Previous studies have barely made comparisons between self-reported and accelerometer-assessed measures of occupational heavy labour. For example, Chau and colleagues [22] were not able to compare self-reported and accelerometer-assessed measures of performing heavy labour, because of the low prevalence of this kind of activities in their mainly office based sample. Likewise, Jancey and colleagues [19], Pedersen and colleagues [23], van Nassau and colleagues [24], and Wick and colleagues [25] did not investigate this correlation either. In this respect, the present study is a valuable addition to the current literature.

The results of a sub-analysis revealed a moderating effect of the work schedule of employees on the association between self-reported and accelerometer-assessed sitting and standing. The concurrent validity of both patterns were lower among shift workers compared to daytime workers. A possible explanation for the lower validity of sitting and standing might be the irregular hours and the lack of a steady pattern in the work schedule of shift workers, which makes it more difficult to report accurately on the conducted activities.

### Under- and overestimations of the OSPAQ

An underestimation of self-reported sitting and standing was observed for workers with physically active professions, whereas for workers with sedentary professions only an underestimation for standing was attested. Similar findings regarding the underestimation of self-reported sitting time in physically active professions were observed in previous studies that used Actigraph accelerometers as a criterion measure [32]. A possible explanation for this finding might be that sitting is less integrated in the daily occupational activities for workers with physically active professions, which might make it more difficult for them to define the time spent sitting. Despite the absence of any underestimation in the sample of workers with a sedentary profession, other studies found an underestimation of the time spent sitting [33–35]. Since most studies have observed an underestimation of self-reported sitting time, the use of a questionnaire to assess occupational sitting time might lead to underestimation of exposure to sitting time and should be interpreted with caution.

Furthermore, participants overestimated self-reported walking in both physically active and sedentary professions, while the time spent performing heavy labour was only overestimated by workers with physically active professions. Our findings are consistent with the findings reported by Ainsworth et al. (1999) [36] from their evaluation of the Tecumseh Self-Administered Occupational Activity Questionnaire (TOQ), which revealed a self-reported overestimation of nearly 10 h per week for total PA at work compared with PA records. A possible explanation for this finding might be that participants with a physically active profession assume that they are active during the whole day, while actually some of their actions are not as active or intensive as they assume, and this might lead to misclassifications of their SB and PA. In addition, social desirability might also play a role in this finding.

### Strengths and limitations

A main strength of this study was the use of accelerometers to assess the concurrent validity of the OSPAQ. Comparisons with accelerometers are considered as an appropriate method for assessing the concurrent validity of PA questionnaires [16, 37]. The use of two Axivitiy AX3 accelerometers has the advantage that it allows to differentiate between and determine different activities (i.e. sitting, standing, and walking) with high specificity and sensitivity [28]. Other previous studies that used for example Actigraph accelerometers [22] were only able to determine light-intensity activity and moderate-intensity, which means that standing and walking are considered as a respectively light- and moderate-intensity activity. In addition, the study of van Nassau and colleagues [24] used an ActiGraph accelerometer as a criterion measure and showed weak validity for occupational sitting and standing because this particular accelerometer was not able to distinguish sitting from standing. Furthermore, an activPAL device was used in this study as a criterion measure. Van Nassau and colleagues [24] suggested that the activPAL inclinometers should be used as a preferred type of measure when determining differences in occupational sitting and standing time. Based on the shortcomings of previous studies, we emphasize that using Axivity AX3 accelerometers is a major strength of this study. Another strength of this study is the inclusion of both workers with sedentary and physically active jobs which was not the case in other studies [22]. Additionally, our sample of participants had a more or less balanced ratio between men and women. The large range of occupational physical activity levels in this study sample allows us to assess the validity of the time spent sitting, but also of the time spent standing, walking, and performing heavy labour. Furthermore, the large sample size of the present study is also an important strength.

The study also has some limitations that should be taken into account. An important limitation is that we were not able to detect upper body movement that is performed while sitting or standing. A second limitation might be that the participants had to estimate their sitting, standing, walking, and heavy labour time in percentage. Although none of the participants included in this study reported problems with estimating percentages, previous research has shown that an assessment of activities might be more exact in terms of absolute time instead of a percentage of time [38]. A third limitation was the use of convenience sampling of volunteers, which might lead to a rather limited representativeness of the sample. Some form of selection bias cannot be ruled out, since the majority of participants were highly educated. However, it should be noted that the participants represented a broad range of professions, including people performing primarily sedentary work, but also workers with strenuously physically active professions, which was an important strength of this validation study and allows the generalizability of our findings. A last limitation is the fact that data from the OSPAQ and accelerometers were not obtained simultaneously. Participants were first asked to fill in the OSPAQ and afterwards the accelerometers were attached to the participants’ thigh and back. Both for the questionnaire and for the accelerometer-assessed measurements, the participants were asked to think of a typical working day and to wear the device on at least 2 consecutive and typical working days. In this regard the time frame of our study is slightly different compared to the typical working day in the last 7 days of other studies validating the OSPAQ.

### Future recommendations

The short format of the OSPAQ makes it suitable to assess occupational sitting and standing in large epidemiological research involving numerous lifestyle behaviours and health outcomes. The questionnaire is short and easy to use. On the basis of the results of this study the OSPAQ can be considered suitable for application in different target groups in a broad range of sedentary and physically active professions. A more sensitive accelerometer-assessed measure should be used to assess walking during work.

Other studies which validated more extended questionnaires, such as the 32-item Tecumseh Occupational Activity Questionnaire, have shown an even lower validity for standing (*r* = 0.27) and walking (*r* = 0.32). This further strengthens the added value of using the OSPAQ due to its higher validity for sitting, standing, and walking and in addition the shorter time needed to complete this questionnaire.

## Conclusion

The findings of this study suggest that the OSPAQ instrument has acceptable measurement properties for assessing the time spent sitting and standing at work for both sedentary and physically active professions. When using the OSPAQ for assessing occupational walking and performing heavy labour, it should be noted that a low to weak concurrent validity was found in the present study and an overestimation of both activities is possible. As a result of the poor concurrent validity for occupational walking and heavy labour, accelerometer-based measures of occupational PA are recommended.

## Supplementary information

**Additional file 1.** Flowchart of the recruitment of the study population; Description: This figure provides more detailed information of the flow of the recruitment of the study population.

**Additional file 2.** Dataset of the study; Description: The data analysed during this study can be found in this additional file. The dataset includes information about the job type of the participants (i.e. 1 = physically active job; 2 = sedentary job), the OSPAQ (i.e. percentage of time spent sitting, standing, walking, and heavy labour during work), and accelerometer data (i.e. percentage of time spent sitting, standing, walking, and heavy labour).

**Additional file 3. Table S3**a: Percentages of self-reported (OSPAQ) and accelerometer-assessed measures of daytime and shift workers separately; Description: Table S3a shows the mean percentages of work time for self-reported and accelerometer-assessed measures of occupational sitting, standing, walking, and heavy labour of the total sample, and separately for day and shift workers. Title: **Table S3b**: Concurrent validity of the OSPAQ compared with accelerometer-assessed measures stratified by work schedule; Description: Table S3b shows the Spearman correlation (r) and intraclass correlations (ICC) between self-reported and accelerometer-assessed measures of occupational sitting, standing, walking, and heavy labour stratified by work schedule.

**Additional file 4.** Bland-Altman plots of the OSPAQ compared to accelerometer-assessed measures for sitting, standing, walking, and heavy labour; Description: the Bland-Altman plots provides the agreement between the time spent in each activity, i.e. sitting, standing, walking, and heavy labour, measured by the OSPAQ and accelerometers among physical active and sedentary professions.

## Data Availability

All data generated or analysed during this study are included in this published article [and its supplementary information files].

## Abbreviations

OSPAQ: Occupational Sitting and Physical Activity Questionnaire
FEPA: Flemish Employees’ Physical Activity
ICC: Intraclass correlation
r: Spearman rho correlation
PA: Physical activity
CVD: Cardiovascular disease
LTPA: Leisure time physical activity
OPA: Occupational physical activity
SB: Sedentary behaviour
BMI: Body mass index
SD: Standard deviation
TOQ: Tecumseh Self-Administered Occupational Activity Questionnaire
