# Supplementary figures and images for: The occupational sitting and physical activity questionnaire (OSPAQ): a validation study with accelerometer-assessed measures
Source: BMC Public Health. 2020 Jul 6;20:1072. doi: 10.1186/s12889-020-09180-9 (PMC7339490; doi:10.1186/s12889-020-09180-9)

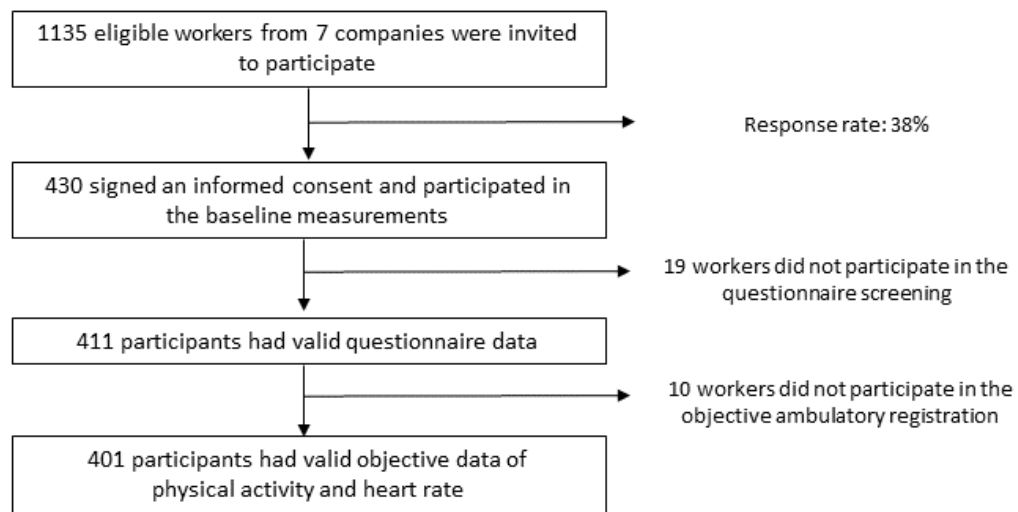

**Figure S1:** Flowchart of the recruitment of the study population

Supplement: Supplementary file 1 — Additional file 1. Flowchart of the recruitment of the study population; Description: This figure provides more detailed information of the flow of the recruitment of the study population. [file 12889_2020_9180_MOESM1_ESM.pdf]
