# Supplementary material for: The occupational sitting and physical activity questionnaire (OSPAQ): a validation study with accelerometer-assessed measures
Source: BMC Public Health. 2020 Jul 6;20:1072. doi: 10.1186/s12889-020-09180-9 (PMC7339490; doi:10.1186/s12889-020-09180-9)
Supplement: Supplementary file 3 — Additional file 3. Table S3a: Percentages of self-reported (OSPAQ) and accelerometer-assessed measures of daytime and shift workers separately; Description: Table S3a shows the mean percentages of work time for self-reported and accelerometer-assessed measures of occupational sitting, standing, walking, and heavy labour of the total sample, and separately for day and shift workers. Title: Table S3b: Concurrent validity of the OSPAQ compared with accelerometer-assessed measures stratified by work schedule; Description: Table S3b shows the Spearman correlation (r) and intraclass correlations (ICC) between self-reported and accelerometer-assessed measures of occupational sitting, standing, walking, and heavy labour stratified by work schedule. [file 12889_2020_9180_MOESM3_ESM.pdf]

Table S3a: Percentages of self-reported (OSPAQ) and accelerometer-assessed measures of daytime and shift workers separately.

	**Total sample (401)**	**Daytime workers (167*)**	**Shift workers (232*)**	**P-value**
**% Sitting: mean (SD)**				
**OSPAQ**	26.34 (29.55)	43.06 (35.62)	14.12 (15.18)	<0.001
**Accelerometers**	34.34 (23.69)	44.46 (28.33)	27.10 (16.38)	<0.001
	<0.001	0.406	<0.001	
**% Standing: mean (SD)**				
**OSPAQ**	28.14 (22.71)	25.67 (25.58)	30.03 (20.32)	0.075
**Accelerometers**	35.38 (17.06)	31.67 (20.13)	38.07 (13.99)	<0.001
	<0.001	0.004	<0.001	
**% Walking: mean (SD)**				
**OSPAQ**	29.90 (18.64)	20.06 (16.64)	35.34 (17.36)	<0.001
**Accelerometers**	15.55 (8.36)	11.67 (6.81)	18.29 (8.30)	<0.001
	<0.001	<0.001	<0.001	
**% Heavy labour: mean (SD)**				
**OSPAQ**	16.68 (18.22)	11.14 (18.78)	20.65 (16.65)	<0.001
**Accelerometers**	13.53 (7.22)	10.27 (6.11)	15.87 (7.08)	<0.001
	0.002	0.806	<0.001	

Data are presented as mean ± SD. *= 2 missings.

Table S3b: Concurrent validity of the OSPAQ compared with accelerometer-assessed measures stratified by work schedule.

	**Total sample (401)**		**Daytime workers (167*)**		**Shift workers (232*)**	
	r	ICC	r	ICC	r	ICC
**% Sitting**	0.69	0.84	0.77	0.88	0.45	0.60
**% Standing**	0.53	0.64	0.60	0.69	0.41	0.55
**% Walking**	0.49	0.50	0.47	0.50	0.29	0.34
**% Heavy labour**	0.35	0.28	0.35	0.24	0.09	0.13

Spearman correlations = r; Intraclass correlations = ICC; *= 2 missings.
