# Supplementary material for: The occupational sitting and physical activity questionnaire (OSPAQ): a validation study with accelerometer-assessed measures
Source: BMC Public Health. 2020 Jul 6;20:1072. doi: 10.1186/s12889-020-09180-9 (PMC7339490; doi:10.1186/s12889-020-09180-9)
Supplement: Supplementary file 4 — Additional file 4. Bland-Altman plots of the OSPAQ compared to accelerometer-assessed measures for sitting, standing, walking, and heavy labour; Description: the Bland-Altman plots provides the agreement between the time spent in each activity, i.e. sitting, standing, walking, and heavy labour, measured by the OSPAQ and accelerometers among physical active and sedentary professions. [file 12889_2020_9180_MOESM4_ESM.pdf]

1a: Sitting time (%) among physically active jobs

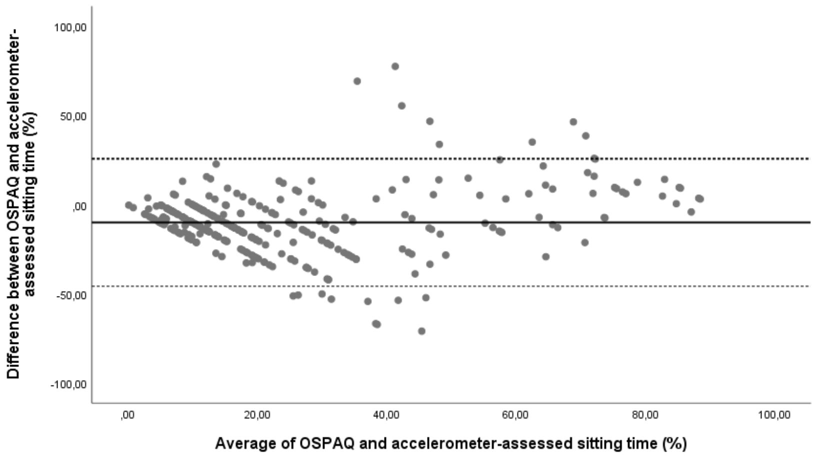

1b: Sitting time (%) among sedentary jobs

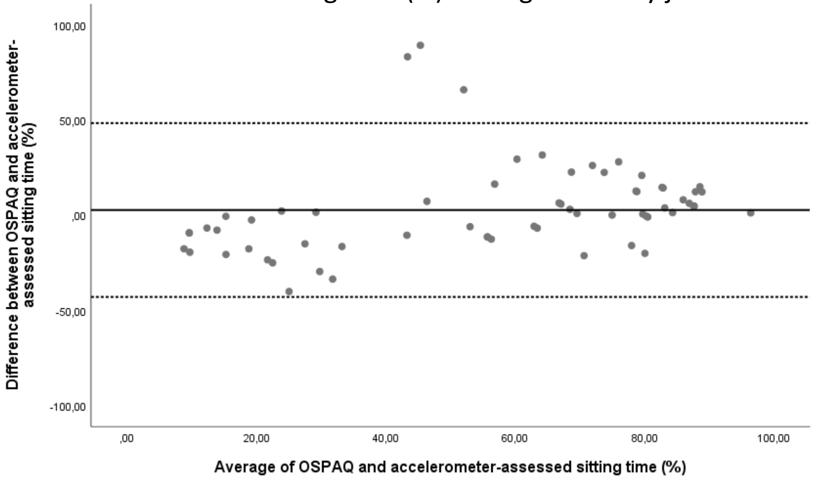

2a: Standing time (%) among physically active jobs

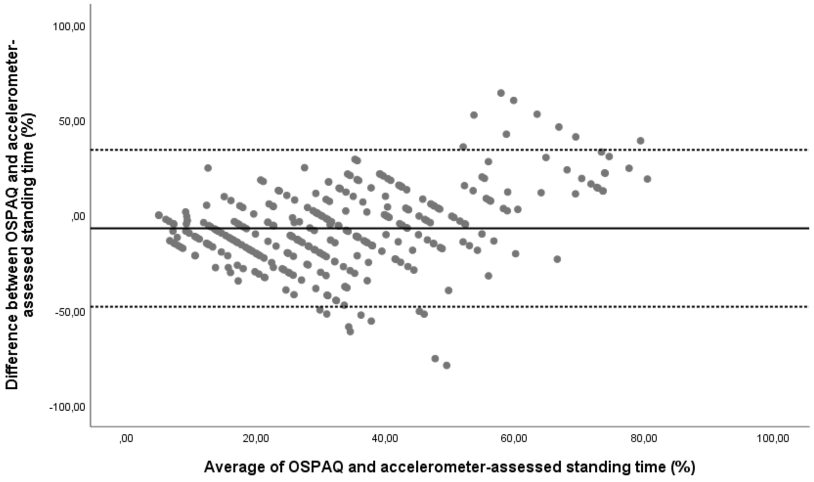

2b: Standing time (%) among sedentary jobs

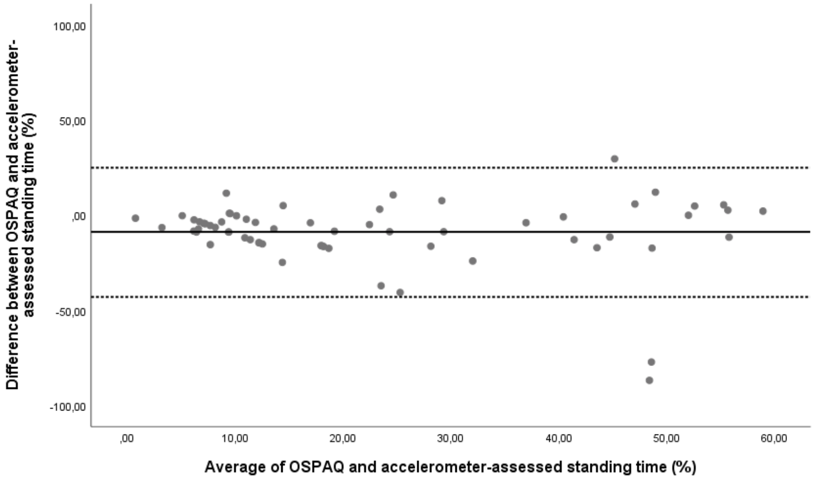

3a: Walking time (%) among physically active jobs

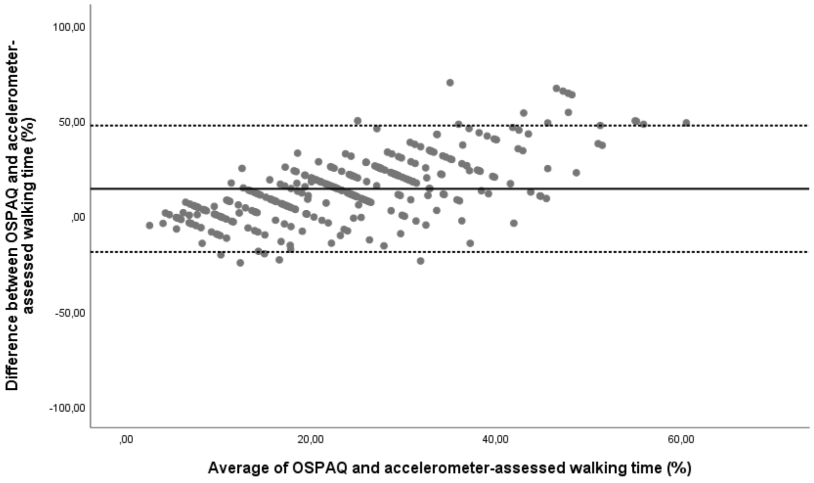

3b: Walking time (%) among sedentary jobs

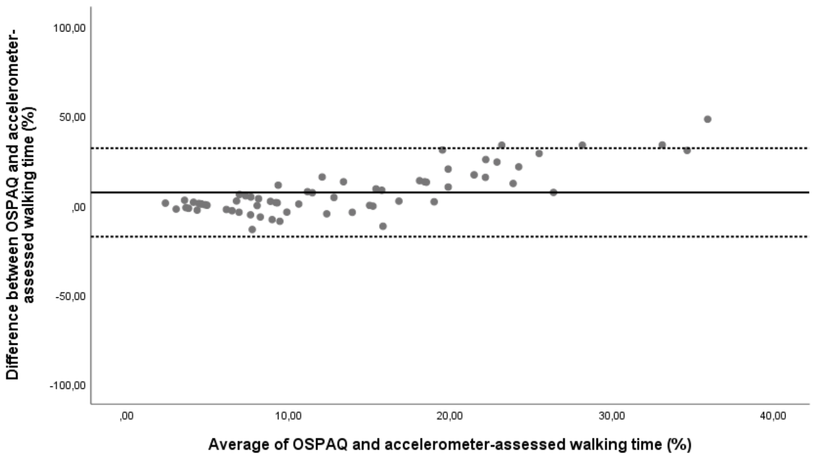

4a: Heavy labour time (%) among physically active jobs

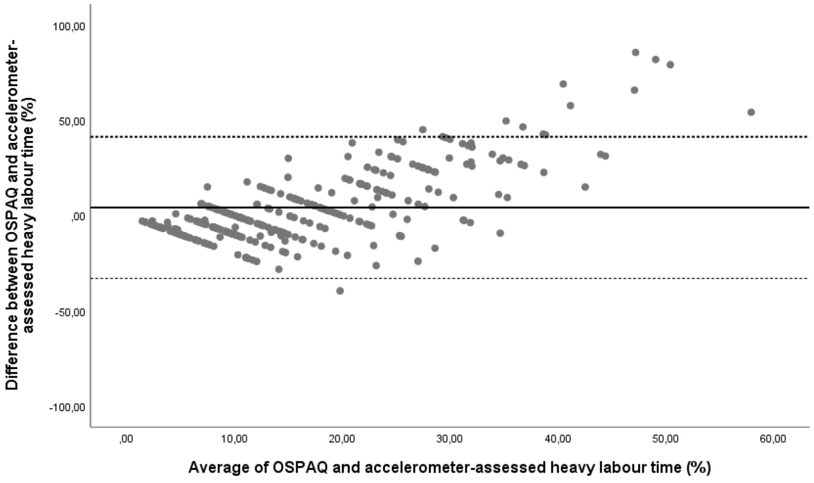

4b: Heavy labour time (%) among sedentary jobs

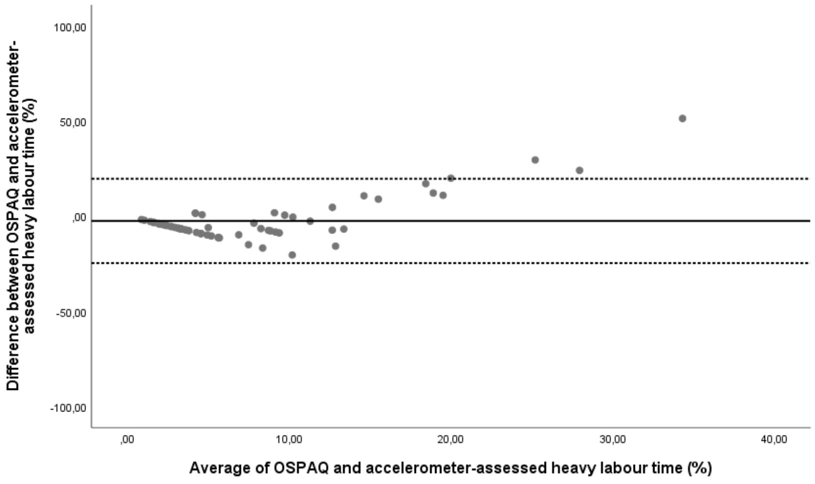

Figure S4: Bland-Altman plots of the OSPAQ compared to accelerometer-assessed (Axivity AX3) measures for sitting (1a & b), standing (2a & b), walking (3a &b), and heavy labour (4a & b) separately for physically active (a) and sedentary jobs (b). The solid line represents the mean difference between the two measures and the dotted line the representation of the limits of agreement, from -1.96sd to +1.96sd.
